# The Effects of Motivational Interviewing on Promoting Human Papillomavirus Vaccination Initiation and Completion Among South Asian Mother/Daughter Dyads: A Pilot Randomised Controlled Trial

**DOI:** 10.1007/s12529-025-10349-y

**Published:** 2025-01-17

**Authors:** Dorothy Ngo Sheung Chan, Kai Chow Choi, Pinky Pui Kay Lee, Winnie Kwok Wei So

**Affiliations:** The Nethersole School of Nursing, The Chinese University of Hong Kong, Hong Kong SAR, China

**Keywords:** Motivational Interviewing, Uterine Cervical Neoplasms, Human Papillomavirus Viruses, Vaccines, Vaccination, Ethnic and Racial Minorities

## Abstract

**Background:**

Vaccination against HPV is an effective strategy for the prevention of HPV infection and cervical cancer. Nevertheless, the HPV vaccine uptake rate is low among ethnic minorities in Hong Kong**.** This study sought to assess the feasibility and acceptability of motivational interviewing among South Asian mother–daughter dyads and to preliminarily examine its effects on knowledge of HPV infection and vaccination, health beliefs, intention to have the daughters vaccinated, and initiation and completion of HPV vaccine series.

**Methods:**

This was a pilot randomised controlled trial. Forty South Asian mothers with at least one daughter aged 9 to 17 years were recruited. The intervention group received a motivational interviewing intervention whereas the control group received usual care. Structured questionnaires were used to collect data on the participants’ characteristics and selected outcome variables. Bias-corrected Hedges’ g and rate difference together with their 95% confidence intervals were calculated to estimate the effect sizes of the intervention on the outcomes The acceptability was assessed via semi-structured interviews.

**Results:**

A larger proportion of the daughters of the intervention group participants had received the first dose of HPV vaccine (95% [19 out of 20]) vs 0% [0 out of 20]). The intervention group showed greater improvement in knowledge at 3 months after the intervention (Hedges’ g = 0.77 (95%CI:0.13–1.41)). Most interviewees were satisfied with the intervention.

**Conclusion:**

The intervention was feasible and acceptable. The intervention can help to increase South Asian mothers’ knowledge and to increase the initiation of HPV vaccine series by their daughters.

**Trial registration:**

This study was registered at the Chinese Clinical Trial Registry (ChiCTR2100052751) on 5 November 2021.

## Introduction

Cervical cancer is a public health concern worldwide and persistent infection with human papillomavirus (HPV) is one of its major causes [1]. In particular, HPV types 16, 18, 31, 33, 45, 52, and 58 account for 90% of cervical cancer cases worldwide. Currently, a nonavalent HPV vaccine is available that provides protection against the most prevalent HPV types. Vaccination is recommended as an effective strategy for the prevention of HPV infection and cervical cancer, especially among adolescent girls aged 9 to 14 years who are advised to receive two doses of the HPV vaccine [2].

Since the introduction of HPV vaccines in 2006, an HPV vaccine has been included in the immunisation programmes of many countries to enhance uptake. According to the National Immune Survey in 2022, 76.0% of adolescents (aged 13 to 17 years) in the United States reported to have received at least one dose of the current HPV vaccine [3]. In Hong Kong, an HPV vaccine was not covered in the routine immunisation programme until 2019 and only Primary 5 and 6 female students can receive the vaccine via this programme [4]. According to a survey conducted by the Family Planning Association in 2021, only 25% of Form 1 to Form 6 female students (aged ~ 12 to 18 years) had received the HPV vaccine [5].

A range of factors guide individuals’ decisions about whether to be vaccinated against a disease. The Increasing Vaccination Model (IVM) as described by Brewer et al. (2017) and Brewer (2021) suggests that people’s thoughts and feelings, such as perceived risk; trust and safety concerns; and social processes, such as provider recommendations and information sharing, can influence the direct behaviour change, that is, their motivation to receive vaccination. Moreover, even when people are motivated to be vaccinated, practical issues, including vaccine availability, may influence whether they take actions that ultimately lead to their being vaccinated [6, 7]. Furthermore, parents are the important persons in the decision-making for an HPV vaccine uptake among adolescents. Parents’ acceptance of HPV vaccination is lower if they have safety and financial concerns and negative beliefs about the vaccine [8]. Furthermore, compared with the general population, ethnic minorities may experience more barriers to vaccination and previous review showed that the HPV vaccine uptake is lower among the ethnic minority populations [9]. For example, a qualitative study conducted in Hong Kong revealed that South Asian mothers experienced more barriers to HPV vaccination than Hong Kong Chinese mothers, such as barriers associated with inadequate knowledge and awareness of cervical cancer, HPV, and the HPV vaccine; negative perceptions about HPV vaccine safety and cost; language difficulties; cultural beliefs; and the absence of family support and healthcare professionals’ recommendations [10].

To encourage mothers to decide in favour of HPV vaccination, interventions should help to address their concerns and enhance vaccine uptake. For example, a previous review revealed that educational interventions can effectively enhance mothers’ knowledge and health beliefs regarding HPV vaccination [11]. It was also found that regular reminders can enhance the completion of HPV vaccine series [11]. However, providing facts about vaccination may not fully satisfy people’s needs, and sending reminders may not necessarily reinforce their decision to be vaccinated. This is because the barriers to vaccination may encompass a set of issues, such as a lack of trust in vaccination, that can also affect people’s motivation to be vaccinated. Thus, work should be done to resolve people’s ambivalence towards or perceived barriers regarding vaccination and to cultivate their motivation to change their beliefs and behaviour.

Motivational interviewing is a client-centred form of communication that may help to motivate people to commit to a behavioural change, as it aims to elicit change by guiding people to explore and resolve their ambivalence [12]. There are four tasks involved in the motivational interviewing, namely engaging, focusing, evoking, and planning, and these tasks enable a client and the motivational interviewing practitioner to engage in a partnership and move towards a commitment to change. A therapeutic relationship is first established during ‘engaging’ task and after that, the purpose of counselling is selected and hence to begin the discussion about behavioural change during the ‘focusing’ task. The practitioner tries to explore and resolve the client’s ambivalence and helps to collect and formulate the reasons for change during the ‘evoking’ task. These tasks also help to foster the client’s motivation or determination for behavioral change. Finally, after the client acquits a readiness for change, appropriate assistance and encouragement are given to help the client to plan and commit to the change during the ‘planning’ task [12].

Motivational interviewing has been adopted in the context of medically related behavioural changes, such as cancer screening and vaccination uptake [13–15].However, few studies using motivational interviewing have been conducted among ethnic minorities to promote HPV vaccination. In Hong Kong, 8.4% of the total population belongs to an ethnic minority. Among this ethnic minority population, South Asians, who mainly originate from India, Pakistan, and Nepal, constitute one of the largest sub-populations [16].In view of the barriers they perceive as affecting their decision making on vaccination, the use of a motivational interviewing intervention could improve their knowledge about HPV vaccines, address their beliefs, resolve their ambivalence, enhance their self-efficacy, and hence increase their initiation and completion of an HPV vaccine series.

The aims of the study were to explore the feasibility and acceptability of motivational interviewing in a sample of South Asian mother–daughter dyads and to preliminarily examine the effects of motivational interviewing on their knowledge and health beliefs, intention to vaccinate, and initiation and completion of HPV vaccine series.

## Methods

### Study Design

This was a pilot randomised controlled trial (RCT) conducted from September 2022 to October 2023. The study was reported according to the Consolidated Standards of Reporting Trials statement [17].

### Participants and Settings

Convenience sampling was adopted, and participants were recruited from ethnic minority community groups that served South Asian populations. The inclusion criteria were (1) a South Asian mother who originated from India, Pakistan, or Nepal; (2) at least one daughter aged 9 to 17 years; (3) the daughter or daughters having no history of HPV vaccination; and (4) the ability to read or communicate in English, Hindi, Urdu, or Nepali. As this study was a pilot RCT, the sample size was not based on hypothesis testing but was expected to be sufficient for assessing the feasibility and preliminarily estimating the effects of the intervention. According to Lancaster et al. [18] a sample size of 30 participants is adequate for an exploratory pilot RCT. By this standard and allowing for an attrition rate of up to 25%, 40 dyads (20 per group) were recruited.

### Randomisation and Blinding

Block randomisation with a block size of eight was used to allocate the participants to either the intervention or control groups. Randomisation was performed using a computer-generated random schedule by a statistician who was not involved in the RCT. A research assistant was responsible for participant recruitment and was blinded to group allocations. After the participants had signed the consent form and completed the baseline questionnaire, they were given sequentially numbered, opaque, sealed envelopes containing group assignments.

### Intervention and Control Groups

Within a week after participant recruitment and the collection of baseline data, the intervention group was provided with the intervention schedule. In addition, a 1-h motivational interviewing session was held with each participant in the intervention group. The sessions were conducted by a motivational interviewing practitioner and ethnically matched, trained community health workers (CHWs). Following the motivational interviewing principle [12], the components of motivational interviewing adopted in the sessions included:

- establishing a rapport with the participants and engaging them in a discussion of cervical cancer and HPV vaccination;
- stating the purposes of the session, which included discussing HPV vaccination;
- helping the participants to explore and resolve their ambivalence towards the HPV vaccine and eliciting talk about change;
- exploring the participants’ readiness for their daughters to receive the HPV vaccine and enhancing their motivation to have their daughters receive the vaccine; and
- providing information about practical issues, and offering logistical assistance as permitted by the participants.

**Fig. 1:**
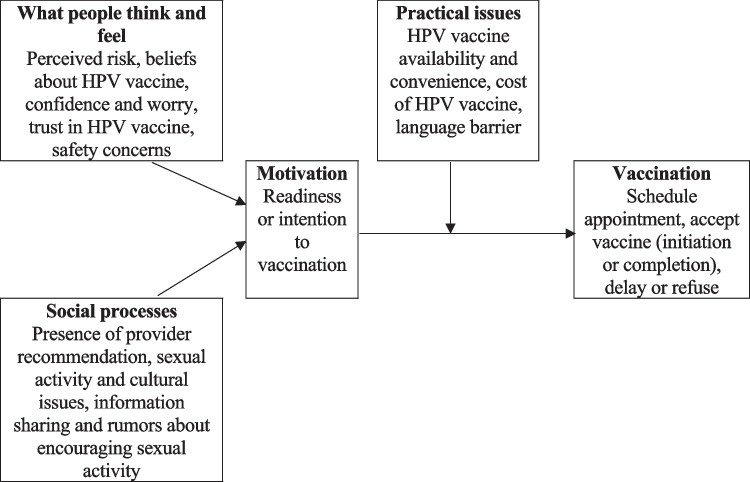
Increasing Vaccination Model focusing on HPV vaccination

The exploration of the participants’ ambivalence and the enhancement of their motivation to undergo vaccination through the provision of informational and logistical support to overcome practical issues were guided by the IVM and previous literature (Fig. 1 and Table 1) [6, 7, 10]. At 1 and 3 months after the motivational interviewing session, the CHWs reminded the participants by telephone or text message to review their daughters’ vaccination statuses. Motivational interviewing was implemented as appropriate during the reminder calls or in the messages.

**Table 1:** Contents Included to Explore and Resolve Ambivalence and Practical Issues with Guidance from the Increasing Vaccination Model

Exploration of ambivalence	Contents included to resolve ambivalence
**What people think and feel:** Beliefs about HPV vaccines and the associated disease (Perceived risk of cervical cancer, perceived benefits of HPV vaccine in offering protection, perceived barriers to HPV vaccination including trust and safety concerns) Confidence and worries about the effect of HPV vaccines **Social processes:** Provider recommendation, sexual activity and cultural issues, sharing of information and rumours about HPV vaccines	• What cervical cancer is, the incidence and mortality, the risk factors and the signs and symptoms • What HPV is and the common HPV types in Hong Kong • What the HPV vaccine is; why HPV vaccination is important; HPV vaccine efficacy, common adverse effects and safety • The importance of vaccination, with or without recommendation • Correction of rumours, including myths and misconceptions about HPV vaccination • Acknowledgement of the cultural issues of South Asians concerning HPV vaccination and the reinforcement of the protection offered by the HPV vaccine
**Existing practical issues**	**Contents included to provide information related to HPV vaccination**
Vaccine availability Cost of HPV vaccine Service providers for HPV vaccination (convenience of obtaining vaccination) Appointment scheduling Language barriers	• Types of available HPV vaccines • Cost of HPV vaccine in the public and private sectors • Service providers and venues for HPV vaccination • HPV vaccination in the Hong Kong Childhood Immunisation Programme • HPV vaccination schedule (two or three doses) and injection route • CHWs will offer help in scheduling appointments and language support during appointment attendance

The participants in the control group received a brief factsheet about vaccination, which is consistent with usual care.

### Outcome Measures

The outcome measures were (1) feasibility of the study; (2) initiation and completion of the HPV vaccine series; (3) intention to have the daughters vaccinated; (4) knowledge about HPV infection and vaccination; (5) perceived susceptibility to and severity of HPV infection or its associated diseases, perceived benefits of and barriers to receiving HPV vaccination; and perceived self-efficacy regarding vaccination; and (6) acceptability of the intervention. Socio-demographic data, such as age, level of education, family income, years lived in Hong Kong, and family history of cancer, were collected at baseline (T0).

The feasibility of the study was assessed by comparing the numbers of dyads who were approached, screened, and considered eligible to join the study, completion of the intervention and follow-up assessments. The number of received HPV vaccine doses was assessed by asking whether participants’ daughters had initiated a vaccine series (≥ 1 dose; ‘Yes’ or ‘No’) and completed the vaccine series (2 or 3 doses, ‘Yes’ or ‘No’). The participants were asked to show their daughters’ vaccination records for verification. Five items were used to assess the participants’ intention to have their daughters vaccinated. These items addressed their previous attempts to obtain information, the extent to which they had considered having their daughters vaccinated, and their likelihood of doing so if the vaccine was offered by their healthcare provider. The items were scored on a 7-point Likert scale (1 = strongly disagree to 7 = strongly agree), where a higher score indicates a higher intention to vaccinate. The scale was confirmed to have good reliability (Cronbach’s α = 0.96) [19]. The short form of the 10-item HPV Knowledge Scale was used to assess the participants’ knowledge of HPV infection and vaccination, with the possible responses being ‘True’ or ‘False’. Each correct and incorrect answer is allocated 1 point and 0 points, respectively, and the points for all answers are summed to give the final score. The scale was shown to exhibit good reliability (Cronbach’s α = 0.85–0.88) [20].

Nine items were used to assess the mothers’ perception of their daughters’ susceptibility to HPV infection and its associated diseases, and nine items were used to assess the mothers’ perception of the severity of an HPV infection and its associated diseases. The items were scored on a 7-point Likert scale (1 = very unlikely to 7 = very likely), where a higher total score indicates that a mother perceives that her daughter is more highly susceptible to HPV infection and its associated diseases or that an HPV infection and its associated diseases are more severe diseases. Both scales were shown to exhibit good reliability (Cronbach’s α = 0.94 (perceived susceptibility); 0.91 (perceived severity)) [19]. Four items were used to assess the participants’ perception of the benefits associated with their daughters being vaccinated against and protected from diseases. Three items were used to assess the participants’ level of confidence in their ability to access vaccination. The items were scored on a 7-point Likert scale (1 = disagree strongly to 7 = agree strongly), where a higher total score indicates that a participant perceives HPV vaccination as more highly beneficial or has higher self-efficacy in accessing vaccination. The scales were shown to have good reliability (Cronbach’s α = 0.88 (perceived benefits); 0.85 (perceived self-efficacy) [19]. Three items were used to assess the perceived barriers to receiving HPV vaccination, namely cost (one item) and safety concerns (two items). The items were scored on a 7-point Likert scale (1 = disagree strongly to 7 = agree strongly), where a higher total score indicates a higher perceived barrier to HPV vaccination. The scale for safety concerns was shown to have good reliability (Cronbach’s α = 0.87) [19].

To determine the acceptability of the intervention, in-depth, semi-structured interviews were conducted via telephone with the intervention group in the languages preferred by the participants. The interviews were conducted by trained interviewers who were proficient in English, Hindi, Urdu, or Nepali. The participants in this group were asked their experience with the intervention. The interviews were approximately 30 min in duration and audio recorded.

### Data Collection Procedure

Ethical approval was obtained from the study institution’s ethics committee (Ref. No.: 2021.434). The research assistant approached potential participants, and those who were eligible were briefed on the study. Written consent was obtained from those who agreed to participate, who were then asked to complete a baseline (T0) questionnaire during a face-to-face interview. At 1 (T1), 3 (T2), and 6 (T3) months after the intervention, the participants completed another questionnaire via a telephone interview. At T0, the questionnaire collected data on the participants’ socio-demographic characteristics. At T0, T1, and T2, the questionnaire collected data on the participants’ knowledge about HPV infection and vaccination, perceived susceptibility to and perceived severity of HPV infection and its associated diseases, perceived benefits of vaccination, perceived barriers to vaccination, perceived self-efficacy regarding vaccination, and intention to have the daughters vaccinated. At T1, T2, and T3, the questionnaire collected data on initiation of the HPV vaccine series, and at T3, it collected data on completion of the HPV vaccine series. Those who had not initiated HPV vaccination were asked to supply their reasons, and these were documented.

### Statistical Analysis

IBM Statistical Products and Service Solutions version 26.0 was used for statistical analysis. The data were summarised and were presented using appropriate descriptive statistics. Normality of continuous variables was assessed based on their skewness and kurtosis statistics and normal probability plots; no continuous variable was found deviated from normality. Given the small sample size of the pilot study, the outcome analysis was focused on effect size estimation rather than statistical significance. Bias-corrected Hedges’ g and rate difference together with their 95% confidence intervals were calculated to estimate the effect sizes of the intervention on the outcomes [21]. The recordings of the interviews were transcribed verbatim by trained bilingual translators. To ensure that the interview dialogues translated into English were consistent with those in the languages used by the participants, the transcripts were validated by other bilingual checkers. A researcher who was competent in English then analysed the collected data using content analysis [22], which corresponded to the objective concerning the acceptability of the intervention.

## Results

### Feasibility of the Study

**Fig. 2:**
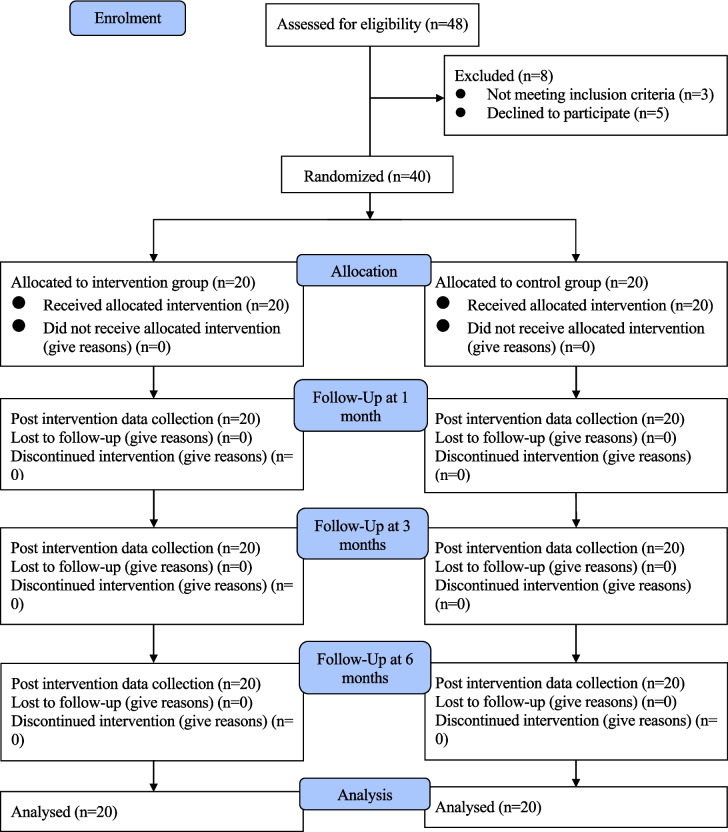
Flow diagram of intervention and data collection points

Forty-eight potential participants were approached. Three potential participants were ineligible because of the age of their daughters. Five potential participants declined to participate due to a lack of interest or time. The remaining 40 participants with age-eligible daughters (14 Indian, 14 Pakistani, and 12 Nepalese women) consented to participate, equating to a consent rate of 88.9%. All participants in the intervention group received and completed the intervention. The intervention completion rate was 100%. All participants completed the follow-up assessments, equating to a retention rate of 100% (Fig. 2).

### Participants’ Characteristics

The mean age of the participants was 40.8 years (standard deviation = 5.9 years), and 95% were married. Approximately 53% had heard of HPV vaccines and 15% had received a recommendation from a doctor regarding HPV vaccination for their daughters (Table 2).

**Table 2:** Baseline Characteristics of the Mother Participants (N = 40)

**Characteristics**	Control (n = 20)	Intervention (n = 20)
Age (years) ^a^	40.8 (5.7)	40.9 (6.1)
Marital Status		
Divorced / separated	1 (5.0%)	1 (5.0%)
Married	19 (95.0%)	19 (95.0%)
Educational level		
Secondary or below	7 (35.0%)	8 (40.0%)
Post-secondary or above	13 (65.0%)	12 (60.0%)
Monthly household income (HK$)		
< 20,000	13 (65.0%)	13 (65.0%)
≥ 20,000	7 (35.0%)	7 (35.0%)
Number of children		
1	3 (15.0%)	4 (20.0%)
2	9 (45.0%)	4 (20.0%)
3	5 (25.0%)	10 (50.0%)
≥ 4	3 (15.0%)	2 (10.0%)
Years of living in Hong Kong		
≤ 10	7 (35.0%)	3 (15.0%)
> 10—20	6 (30.0%)	10 (50.0%)
> 20	7 (35.0%)	7 (35.0%)
Family history of cervical cancer		
No	20 (100.0%)	19 (95.0%)
Yes	0 (0.0%)	1 (5.0%)
Health insurance		
No	15 (75.0%)	16 (80.0%)
Yes	5 (25.0%)	4 (20.0%)
Heard about HPV vaccine before		
No	13 (65.0%)	8 (40.0%)
Yes	7 (35.0%)	12 (60.0%)
Received recommendation from a doctor regarding HPV vaccination for your daughter		
No	18 (90.0%)	16 (80.0%)
Yes	2 (10.0%)	4 (20.0%)
Social norms regarding HPV vaccination [possible score range: 8 – 40] ^a^	22.3 (4.7)	27.0 (3.8)

Variables marked with ^a^ are presented as mean (standard deviation), otherwise as frequency (%)

### Effects of the Intervention on Initiation and Completion of HPV Vaccination

At T1 to T3 and compared with the control group, a larger proportion of the daughters of the intervention group participants had received the first dose of the HPV vaccine [T1: 55% [11 out of 20] vs 0% [0 out of 20], rate difference = 0.55 (95% confidence interval (CI): 0.25–0.76); T2: 75% [15 out of 20]) vs 0% [0 out of 20], rate difference = 0.75 (95% CI: 0.43–0.90); T3: 95% [19 out of 20]) vs 0% [0 out of 20], rate difference = 0.95 (95% CI: 0.65–1.00)] (Table 3). At T3, five daughters of the intervention group participants had received all the required doses of the HPV vaccine. Common reasons for not receiving the HPV vaccine were its cost, preference to wait to receive it later from their school, and fear of side effects.

**Table 3:** Outcome Measures across Study Time Points between the Two Groups (N = 40)

		Control group	Intervention group	Effect size (95% CI)
**Outcomes**		n (%) /Mean (SD)	n (%) /Mean (SD)	
Initiation of HPV vaccination ^a^	T1	0 (0.0%)	11 (55.0%)	0.55 (0.25, 0.76) ^b^
	T2	0 (0.0%)	15 (75.0%)	0.75 (0.43, 0.90) ^b^
	T3	0 (0.0%)	19 (95.0%)	0.95 (0.65, 1.00)^b^
Intention to get vaccinated for HPV	T0	4.72 (1.49)	5.99 (0.77)	
[Possible score range: 1 – 7]	T1	5.19 (1.21)	6.57 (0.40)	0.10 (−0.52, 0.72)^c^
	T2	5.49 (1.08)	6.73 (0.36)	−0.02 (−0.64, 0.60)^c^
Knowledge about HPV infection and	T0	2.30 (2.32)	2.95 (2.09)	
Vaccination	T1	4.15 (3.22)	6.45 (2.58)	0.46 (−0.16, 1.09)^c^
[Possible score range: 0 – 10]	T2	4.60 (3.08)	7.85 (2.16)	0.77 (0.13, 1.41)^c^
Perceived susceptibility	T0	3.76 (1.67)	4.81 (1.07)	
[Possible score range: 1 – 7]	T1	3.98 (1.58)	5.13 (1.04)	0.10 (−0.52, 0.73)^c^
	T2	4.57 (1.26)	5.54 (0.96)	−0.07 (−0.69, 0.55)^c^
Perceived severity	T0	3.87 (2.02)	5.27 (0.85)	
[Possible score range: 1 – 7]	T1	5.27 (1.13)	5.91 (0.95)	−0.49 (−1.12, 0.14)^c^
	T2	5.97 (0.79)	6.21 (0.95)	−0.64 (−1.27, 0.00)^c^
Perceived benefit	T0	5.26 (1.15)	6.10 (0.67)	
[Possible score range: 1 – 7]	T1	5.74 (0.95)	6.45 (0.50)	−0.13 (−0.75, 0.49)^c^
	T2	5.88 (0.88)	6.75 (0.40)	0.03 (−0.58, 0.65)^c^
Perceived barriers – cost	T0	4.25 (2.20)	4.35 (2.03)	
[Possible score range: 1 – 7]	T1	4.35 (1.90)	4.00 (2.27)	0.26 (−0.36, 0.89)^c^
	T2	4.20 (1.99)	3.50 (2.21)	0.44 (−0.19, 1.07)^c^
Perceived barriers – safety concerns	T0	5.05 (1.60)	4.80 (1.38)	
[Possible score range: 1 – 7]	T1	4.40 (1.60)	4.33 (1.93)	−0.10 (−0.72, 0.52)^c^
	T2	4.33 (1.43)	4.30 (1.95)	−0.14 (−0.76, 0.48)^c^
Perceived self-efficacy	T0	5.13 (1.12)	5.95 (0.79)	
[Possible score range: 1 – 7]	T1	5.52 (0.70)	6.33 (0.65)	0.00 (−0.62, 0.62)^c^
	T2	6.05 (0.67)	6.37 (0.81)	−0.40 (−1.03, 0.23)^c^

Variables marked with ^a^ are presented as frequency (%), otherwise as mean (standard deviation)

^b^ Rate difference

^c^ Hedges’ g effect size which corresponds to the standardized mean difference of the mean changes at the underlying time point with respect to T0 between the intervention and control groups

### Effects of the Intervention on Knowledge, Health Beliefs and Intention to get HPV Vaccination

Table 3 shows the outcome measures of knowledge, health beliefs and intention to get HPV vaccination across the study time points in both groups. At T0, the groups had similar knowledge about HPV infection and vaccination and perceived barriers to vaccination (cost and safety concerns) but differed in their intention to have their daughters vaccinated, perceived susceptibility to and perceived severity of HPV infection and its associated diseases, and perceived benefits of and self-efficacy regarding HPV vaccination.

At T1, there were no notable difference in changes in outcome measures between the two groups. Nevertheless, compared with the control group, the intervention group showed a greater trend of improvement in some outcome measures, namely intention to have the daughters vaccinated, knowledge about HPV infection and vaccination, perceived susceptibility to HPV infection and its associated diseases, and perception of cost as a barrier to vaccination. At T2 and compared with the control group, the intervention group showed a notably greater increase in knowledge about HPV infection and vaccination (Hedges’ g = 0.77 (95% CI: 0.13–1.41)). A notably greater change in the perceived severity of the disease was observed in the control group than that of the intervention group (Hedges’ g = −0.64 (95% CI: −1.27–0.00)). At T2, there were no notable difference of changes between groups in other outcome measures.

### Acceptability of the Intervention

Twenty participants in the intervention group were interviewed. Four categories were generated regarding the acceptability of the intervention and their experience of participation: (1) enlightened conversation that increased understanding of HPV vaccination; (2) embracing decision making with confidence; (3) support from the team; and (4) areas for improvement.

Enlightened conversation that increased understanding of HPV vaccination

The interviewees revealed that they had good experiences as participants. The process of conversation between the motivational interviewing practitioner, CHWs, and the participants was collaborative, and explored and resolved the participants’ ambivalence regarding vaccination, which had affected their consideration of whether to receive HPV vaccination.

*‘I went there with my questions, and they gave me clear answers.’ (P14)*

*‘I was happy as I was able to ask questions about the things that I did not understand. I got to know more and understood [about HPV vaccination] in this session. The best part was being able to have a two-way conversation and ask questions.’ (P18)*

The interviewees appreciated the opportunity to obtain clear and helpful information about the HPV vaccine. They learned about the potential benefits of the HPV vaccine and understood that having their daughters vaccinated would help to safeguard their health by reducing their risk of developing cervical cancer later in life.

*‘I learned that it would be good to get my daughter vaccinated, as it will keep her safe from the cancer caused by that virus.’ (P15)*

*‘I was told by some of my friends that it’s very important for girls to get the HPV vaccination when they are young. I didn’t know how to approach this and whom to approach about this. I got very good information from your team.’ (P06)*

The interviewees did have doubts and concern about the HPV vaccine. They wanted to ensure that the HPV vaccine was suitable for their daughters and to clarify their understanding about long-term side effects before they made their decision.

*‘The school just sends us the form. They never explain the (HPV vaccination) benefits and risks; they just tell you that you should get it. However, at the interview session, they explained the information well. I now completely understand why we should get [the HPV vaccine].’ (P10)*

*‘I couldn’t understand why it was necessary because generally people don’t get vaccinated unless there is something like COVID-19 around. The session helped me to understand that [the HPV vaccine] is a preventive measure. I learnt a lot and it helped to get rid of all my doubts and misconceptions.’ (P06)*

Embracing decision making with confidence

The conversation in the motivational interviewing session had a positive impact on the decision-making process of the interviewees. They agreed that it was important to have access to accurate and detailed information to support one’s ability to make informed decisions.

*‘I got to know the reason for vaccination. Many people may get their daughters vaccinated without knowing all of the details. I got the chance to learn these details and clarify them with you. This greatly affected my decision.’ (P18)*

*‘Being a mother, I need to do this to protect my daughter. I have finally decided that it is okay to now take the next step and have my daughter vaccinated.’ (P08)*

The motivational interviewing session not only contributed to the decision-making process of the interviewees but also to that of their daughters. The interviewees also shared the information with their husbands and received support from them.

*‘After we had the session, I discussed it with my daughter and she said “Mummy, let’s do it. That’s good for health.” I then talked to my husband and daughter about [HPV vaccination], and we decided that we would go for vaccination.’ (P10)*

*‘[My daughter] was well informed about [HPV vaccination] and why she was being vaccinated, and how it will protect her in the future. She agreed to do this. This is the main thing you learn: that they need to agree and ultimately it’s their body.’ (P07)*

*Support from the team*

The interviewees expressed their appreciation for the team’s dedication to providing culturally sensitive care to enhance HPV vaccine uptake. They appreciated the team’s effort in conducting the session and providing invaluable information and support. In addition, the session was conducted in their language with the support of the CHWs, thereby fostering good understanding and enabling the interviewees to effectively grasp the details.

*‘They arranged an interpreter and so everything was well explained. At school, [the teachers] speak in English and Chinese, so we don’t [understand]. Vaccination programmes should be in our language, so we can clearly understand their purpose and not panic about them.’ (P10)*

Moreover, the interviewees stated that the team provided information to them about a clinic that offered HPV vaccination services that catered to the needs of ethnic minorities. They saw that this demonstrated a commitment to equitable healthcare access and an understanding of the unique challenges faced by ethnic minorities.

*‘It was quite easy, and the process [from attending the session to receiving vaccination] was smooth. Also, the clinic was in an accessible location, and the nurses in the clinic were good. They explained the whole process to us before they gave the vaccination to my daughter.’ (P03)*

*Areas for improvement*

All interviewees commented that the programme was good but noted that such programmes should be promoted widely, to ensure that more ethnic minority people, especially those who have daughters, can benefit and receive the necessary support.

*‘More outreach for this programme would be very helpful. The local Chinese population can get to know about [the HPV vaccine] from the local media or newspaper. But ethnic minorities [such as us] sometimes feel a bit left out of all these facilities/programmes organised by the government.’ (P06)*

## Discussion

This study assessed the feasibility and acceptability of a motivational interviewing intervention among South Asian women and its preliminary effects on selected outcomes. The consent to participate rate (89%) and the intervention and follow-up assessment completion rates (100%) were high and similar to those in previous studies conducted among South Asian people on cervical and colorectal cancer screening [23, 24]. In addition, the completion rate was higher than that in a similar study that used motivational interviewing among Chinese men to promote HPV vaccination (76%) [25]. The high consent and completion rate of the present study could be related to the strategies used. For example, the research assistant and CHWs were ethnically matched to the potential participants and could speak their languages, which helped participant recruitment and supported intervention implementation [26]. As has been suggested by other studies, it is important to understand the roles and responsibilities of South Asian women at home and in their daily routines. For example, South Asian mothers are available when their children are at school or interest classes [23, 26]. Thus, our data collectors made use of the participants’ free time to complete the follow-up assessments.

User experiences provide valuable feedback for improvement of an intervention. The participants in the present study appreciated the fact that the intervention helped to improve their understanding about the need for HPV vaccination and removed their doubts and concerns about the HPV vaccine. This is consistent with the findings of a previous study on motivational interviewing intervention that aimed to promote colorectal cancer screening among older Chinese adults [27]. A motivational interviewing session provides an opportunity for participants to express their concerns freely, without judgement from others. In addition, a motivational interviewing practitioner answers participants’ questions and meets their needs without giving unnecessary information. During this process, both parties work collaboratively to explore and resolve participants’ ambivalence about the matter of concern. The practitioner helps participants to gather necessary information and develop their arguments. This process empowers participants to make decisions that align with their values and priorities [12]. Furthermore, consistent with previous studies, the participants valued the support offered by the team in relation to where to receive the vaccine and how to obtain language support throughout the process [23, 24]. Language barriers are a common problem experienced by South Asians, as they are not all proficient in using English and Cantonese, the languages commonly used in Hong Kong [10]. For example, South Asian mothers previously mentioned that they often received notices from school that were written in English and Chinese, which they could not read [10]. Similar to previous studies, with the support of the CHWs, the mothers could easily express their concerns in their own languages and understand information provided in their own languages [10, 23]. Thus, in the future, such a programme could be promoted widely, such that more ethnic minority people, especially those who have daughters, could benefit and receive necessary support. Moreover, as all Primary 5 and 6 female students can receive the vaccine for free via the Childhood Immunisation Programme [4], thus, it would be good one or more sessions using motivational interviewing with the support of CHWs could be conducted for mothers of these students at 3 to 6 months before their daughters receive the HPV vaccine at school.

Consistent with a previous study, the intervention group demonstrated increased knowledge about HPV infection and vaccination after they had received the intervention [14]. A previous qualitative study revealed that a lack of knowledge about HPV-related diseases and the HPV vaccine was a major barrier affecting South Asian mothers’ decision to have their daughters vaccinated [10]. In the current study, despite 60% of the intervention group having heard about the HPV vaccine, they did not clearly understand how the vaccine worked and were concerned about its side-effects. As their daughters were aged 9 to 17 years, they needed their mothers’ consent before receiving vaccination [10]. The motivational interviewing practitioner answered the participants’ questions and collaborated with the participants to explore and resolve their conflicted feelings about HPV vaccination for their daughters [10, 12]. Unlike a previous study that found no significant change in HPV vaccine uptake in an intervention group [25], the present study found a notably higher vaccine uptake among the daughters of the mothers in the intervention group than in the control group. These findings suggest that changes in knowledge help mothers to appreciate the benefits of the HPV vaccine, namely its ability to protect their daughters from diseases [10, 12, 27, 28]. In addition, some of the participants mentioned that they had shared the knowledge gained with their husband and daughters and discussed HPV vaccination with them. This highlights that family support is regarded as important by South Asian women when they make a healthcare decision. Positive responses from family members strengthen mothers’ intention to get their daughters vaccinated [10, 28].

Previous studies have found that South Asian mothers are aware the severe consequences of HPV infection and cervical cancer [10, 28]. In the current study, compared with the intervention group, the control group showed a notably greater increase in their perceived severity of the diseases. This increase was not observed in the intervention group because the intervention participants had their concerns or misconceptions assuaged by the tailored information they received during the motivational interviewing session. This diminished their concerns about the consequences of HPV infection and vaccination and that their daughters had received the first dose of the HPV vaccine already [25]. There were no notable between-group differences in several outcomes, namely intention to have the daughters vaccinated, perceived susceptibility to HPV infection and its associated diseases, perceived benefits of vaccination, perceived barriers to vaccination, and perceived self-efficacy in receiving vaccination, which is consistent with the findings of a previous study [25]. However, a trend of improvement was observed in some outcomes. The perception of cost being a barrier to vaccination decreased more in the intervention group than in the control group. This might have been because some daughters of the participants could receive the vaccine for free via the childhood immunisation programme [4]. Those who were not eligible to join this immunisation programme could receive the vaccine at a reasonable price at a clinic supporting ethnic minorities in Hong Kong, and such information was offered during the motivational interviewing session [10]. It was observed that three quarters of the daughters of the participants had received the first dose of the HPV vaccine at T2. Overall, these results suggest that by T2, most of the participants had had their daughters start the vaccination series. Thus, the participants’ perceived that their daughters had decreased susceptibility to HPV infection and its associated diseases [10, 24, 27].

There were some limitations to this pilot RCT. First, the participants were recruited via convenience sampling. Thus, there might have been selection bias that weakened the representativeness of the sample. Second, it was impossible to blind the staff who were helping to deliver the intervention. Third, the follow-up assessments were conducted until only 6 months after the intervention, which meant that information about receiving all doses of the HPV vaccine could not be captured as some of the participants received the second dose a year later. In a future study, a longer (e.g., 12-month) follow-up assessment should be adopted. Fourth, only mothers’ characteristics and responses, not those of the daughters, were collected in this study. The teenagers’ or daughters’ characteristics, such as age, should be considered in future studies. This is important because such characteristics may affect teenagers’ ability and/or willingness to receive information about HPV and HPV vaccines. Lastly, as this was a pilot RCT, the sample size was small. Some non-significant trends in the results observed in this study might be attributable to the small sample size. Thus, a full-scale study is warranted to reveal the effects of the intervention on the selected outcomes. Furthermore, although our study design was an RCT, some between-group differences were observed at baseline in some potential confounding/prognostic factors related to the study outcomes, such as ‘social norms regarding HPV vaccination’ and ‘heard about HPV vaccine before’. The study findings may be confounded by such factors and therefore should be interpreted with caution.

## Conclusion

The current study found that the motivational interviewing intervention was found to be feasible and acceptable to South Asian mothers. The findings suggest that the intervention can help to improve South Asian mothers’ knowledge about the HPV infection, the HPV vaccine, and the initiation of HPV vaccine series by their daughters. Full-scale RCTs are needed to confirm its effects.

## Data Availability

The data that support the findings of this study are available from the corresponding author, upon reasonable request.

## Declarations

### Welfare of Animals

This article does not contain any studies with animals performed by any of the authors.
